# Network Pharmacology Databases for Traditional Chinese Medicine: Review and Assessment

**DOI:** 10.3389/fphar.2019.00123

**Published:** 2019-02-21

**Authors:** Runzhi Zhang, Xue Zhu, Hong Bai, Kang Ning

**Affiliations:** School of Life Science and Technology, Huazhong University of Science and Technology, Wuhan, China

**Keywords:** network pharmacology, Traditional Chinese Medicine, database, assessment and comparison, completeness

## Abstract

The research field of systems biology has greatly advanced and, as a result, the concept of network pharmacology has been developed. This advancement, in turn, has shifted the paradigm from a “one-target, one-drug” mode to a “network-target, multiple-component-therapeutics” mode. Network pharmacology is more effective for establishing a “compound-protein/gene-disease” network and revealing the regulation principles of small molecules in a high-throughput manner. This approach makes it very powerful for the analysis of drug combinations, especially Traditional Chinese Medicine (TCM) preparations. In this work, we first summarized the databases and tools currently used for TCM research. Second, we focused on several representative applications of network pharmacology for TCM research, including studies on TCM compatibility, TCM target prediction, and TCM network toxicology research. Third, we compared the general statistics of several current TCM databases and evaluated and compared the search results of these databases based on 10 famous herbs. In summary, network pharmacology is a rational approach for TCM studies, and with the development of TCM research, powerful and comprehensive TCM databases have emerged but need further improvements. Additionally, given that several diseases could be treated by TCMs, with the mediation of gut microbiota, future studies should focus on both the microbiome and TCMs to better understand and treat microbiome-related diseases.

## Opportunities and Challenges for the Modernization of Traditional Chinese Medicine

### Traditional Chinese Medicine

Traditional Chinese Medicine (TCM) is one of the greatest treasures of Chinese culture, has a long history of use in East and Southeast Asia, and has been widely used since ancient civilization. Through continuous development and innovation, physicians have selected the essence and discarded the dross of TCM. As a result, TCM has become one of the main forms of alternative medicine in East Asia, North America, and Europe. TCMs use therapeutic herbs to treat diseases according to the combinatorial principle of “King, Vassal, Assistant, and Delivery servant” based on a patient’s syndrome and to restore balance of life and body functions (Yi and Chang, 2004; Qiu, 2007). Each prescribed combination of these herbs is referred to as a TCM preparation, or TCM prescription, for example the LiuWeiDiHuangWan (LWDHW) pill. Traditionally, the discovery of most new drugs is focused on identifying or designing a pharmacologically effective agent that specifically interacts with a single target. During the past 30 years, such an approach has generated highly successful drugs. However, drugs acting on individual molecular targets usually exert unsatisfying therapeutic effects or have toxicity when used to treat certain diseases, such as diabetes, inflammation, and cancer (Kola and Landis, 2004). Given the major bottleneck in drug discovery, drug research, and development have gradually shifted from a “one-target, one-drug” mode to a “network-target, multiple-component-therapeutics” mode (Li, 2011; Li et al., 2011, 2014b). To treat disease, the holistic philosophy of TCM shares key concepts with the new mode of drug discovery. Therefore, making full use of TCM is of great importance for drug research. In recent years, TCM has gradually garnered interest thanks to its low toxicity and therapeutic effects (Chan, 1995; Corson and Crews, 2007; Qiu, 2007). For example, *Ganoderma lucidum*, termed “LingZhi” in China, has been used as a health-preserving and therapeutic agent. Previous studies have shown that it possesses medicinal properties, including anti-cancer, anti-diabetic, anti-hepatotoxic, and immunomodulatory effects (Paterson, 2006; Boh et al., 2007, Boh, 2013; Ma et al., 2015). *Panax ginseng* is another TCM that has been used therapeutically for more than 2,000 years to treat such diseases as cardiovascular disease, Alzheimer’s disease, and diabetes (Lee et al., 2008; Kim, 2012; Yuan et al., 2012; Chan et al., 2013). Unlike modern drugs discovered by targeting a specific protein, however, the understanding of the molecular basis of TCM remains limited, and research into modern TCM theory has lagged, which has slowed down the development of novel TCMs (Corson and Crews, 2007). TCM studies must keep pace with the rapid development of modern science to remain relevant. Therefore, a scientific and effective assessment system needs to be established, which will be key for studying and making full use of TCMs.

### Network Pharmacology: An Appropriate Approach for Modern TCM Research

**FIGURE 1:**
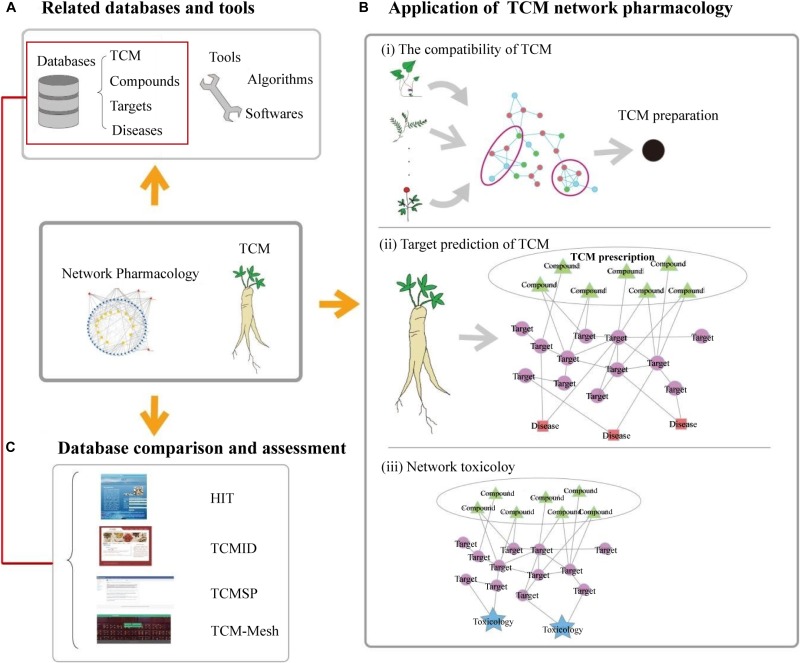
Overall schema of the databases, tools, and applications of TCM network pharmacology analysis. **(A)** Related databases (TCM, compounds, targets, and disease databases) and tools (algorithms and software) used for TCM network pharmacology research. **(B)** The application of network pharmacology to TCM research: (i) the compatibility of TCM; (ii) target prediction of TCM; and (iii) network toxicology. **(C)** Comparison among several TCM databases based on the general statistics and search results.

Given the rapid progress in bioinformatics, systems biology, and polypharmacology, network-based drug discovery is considered to be a promising approach for cost-effective drug development. Systems biology examines biological systems by systematically perturbing them; monitoring the gene, protein, and informational pathway responses; integrating these data; and, ultimately, formulating mathematical models to describe the structure of the system and its response to individual perturbations (Ideker et al., 2001). Based on a systems biology approach, the concept of network pharmacology was first proposed by Li et al. (2014b). Because network pharmacology can provide a full or partial understanding of the principles of network theory and systems biology, it has been considered the next paradigm in drug discovery (Hopkins, 2008). Furthermore, the network pharmacology approach has been used to study “compound-proteins/genes-disease” pathways, which are capable of describing complexities among biological systems, drugs, and diseases from a network perspective, sharing a similar holistic philosophy as TCM. Applications of systems biology methods to determine the pharmacological action, mechanism of action, and safety of TCMs are invaluable for modern research and development of TCM. Thus, a new interdisciplinary method termed TCM network pharmacology has been proposed (Li and Zhang, 2013; Liang et al., 2014a; Li et al., 2014b), which has initiated a new research paradigm for transforming TCM from an experience-based to evidence-based medicine. In this work, we first summarized the currently widely used databases and tools for TCM network pharmacology research. Second, we concentrated on the different applications of network pharmacology to TCM research, including TCM recipes, target prediction, and network toxicology. Third, we compared different TCM databases based on their basic properties and search results (Figure 1).

## Tools and Databases for TCM Network Pharmacology Research

With the rapid development of research, network pharmacology has become a new approach for drug mechanism research and drug development. In recent years, a variety of related databases and tools have provided crucial support for TCM network pharmacology research. Commonly used databases for TCM network pharmacology research include TCM databases [TCM database@Taiwan (Chen, 2011), HIT (Ye et al., 2011), TCMSP (Ru et al., 2014), and TCMID (Xue et al., 2013)), compound and drug information databases (Drugbank (Wishart et al., 2006), STITCH (Kuhn et al., 2014), ChEMBL (Gaulton et al., 2012), and PubChem (Wang et al., 2009)], target interaction databases [STRING (Szklarczyk et al., 2015), HPRD (Peri et al., 2003), MINT (Zanzoni et al., 2002), IntAct (Kerrien et al., 2012), Reactome (D’Eustachio, 2009), and HAPPI (Chen et al., 2009)], and gene-disease association databases [OMIM (Hamosh et al., 2002) and GAD (Becker et al., 2004; Table 1)]. Recently, we also proposed TCM-Mesh (Zhang et al., 2017), a more comprehensive TCM database that embodies the core idea of TCM network pharmacology. Based on these biological databases and clinical trial results, researchers can analyze the “herbs-compounds-proteins/genes-diseases” interaction network from the perspective of systems biology, which will provide an understanding of the effects of herbs on diseases. Furthermore, network pharmacology algorithms and tools are particularly vital in mining these databases for knowledge. For example, the Random Walk (Chen et al., 2012) algorithm is a commonly used network clustering algorithm, which starts from a random node (drug, target, or disease) and calculates the similarity of this node and its adjacent node to construct a “drug-target-disease” network. Moreover, the PRINCE (Vanunu et al., 2010) algorithm is used to prioritize disease genes and infer protein complex associations, based on formulating constraints on the prioritization function which is related with its smoothness over the network and usage of prior information. In addition to data acquisition and analysis, visualization is a key element of network pharmacology that makes the network more intuitive. Cytoscape (Shannon et al., 2003) is an open-source platform suitable for visualizing molecular interaction networks and biological pathways and integrating these networks with annotations, gene expression profiles, and other state data. Pajek (Dohleman, 2006) is another powerful network analysis tool that is used to examine various complex non-linear networks.

**Table 1:** Public databases, algorithms, and software related to TCM network pharmacology.

Type		Name	Description	Website for database or tool	Reference
**Databases**	**TCM-related databases**	TCM-Mesh	An integration of database and a data-mining system for network pharmacology analysis of TCM preparations	http://mesh.tcm.microbioinformatics.org	Zhang et al., 2017
		TCM database@Taiwan	The world’s largest and most comprehensive free small molecular database on TCM for virtual screening	http://tcm.cmu.edu.tw	Chen, 2011
		HIT	A comprehensive and fully curated database to complement available resources on protein targets for FDA-approved drugs as well as the promising precursor compounds	http://lifecenter.sgst.cn/hit	Ye et al., 2011
		TCMSP	A unique systems pharmacology platform of TCMs that captures the relationships among drugs, targets, and diseases	http://lsp.nwu.edu.cn/tcmsp.php	Ru et al., 2014
		TCMID	A comprehensive database that provides information and bridges the gap between TCM and modern life sciences	http://www.megabionet.org/tcmid	Xue et al., 2013
	**Drug-related databases**		A unique bioinformatics and cheminformatics resource that combines detailed drug data with comprehensive drug target information	https://www.drugbank.ca	Wishart et al., 2006
		STITCH	A database of known and predicted interactions between chemicals and proteins	http://stitch.embl.de	Kuhn et al., 2014
		ChEMBL	An Open Data database containing binding, functional, and ADMET information for a large number of drug-like bioactive compounds	https://www.ebi.ac.uk/chembl	Gaulton et al., 2012
		PubChem	A public information system for analyzing the bioactivities of small molecules	https://pubchem.ncbi.nlm.nih.gov	Wang et al., 2009
	**Target-related databases**	STRING	A database of known and predicted protein-protein interactions	https://string-db.org	Szklarczyk et al., 2015
		HPRD	An object database that integrates a wealth of information relevant to the function of human proteins in health and disease	http://www.hprd.org	Peri et al., 2003
		MINT	A database that focuses on experimentally verified protein-protein interactions mined from the scientific literature by expert curators	https://mint.bio.uniroma2.it	Zanzoni et al., 2002
		IntAct	A freely available, open-source database system and analysis tool for molecular interaction data	https://www.ebi.ac.uk/intact	Kerrien et al., 2012
		Reactome	A free, open-source, curated, and peer-reviewed pathway database	https://reactome.org	D’Eustachio, 2009
		HAPPI	An online database of comprehensive human annotated and predicted protein interactions	http://discovery.informatics.uab.edu/HAPPI	Chen et al., 2009
	**Disease-related databases**	OMIM	A comprehensive, authoritative compendium of human genes and genetic phenotypes that is freely available and updated daily	https://www.omim.org	Hamosh et al., 2002
		GAD	A database of genetic association data from complex diseases and disorders	https://geneticassociationdb.nih.gov	Becker et al., 2004
**Algorithms**		Random walk	An algorithm that predicts potential drug-target interactions on a large scale under the hypothesis that similar drugs often target similar target proteins and the framework of Random Walk	https://www.rdocumentation.org/packages/diffusr/versions/0.1.4/topics/random.walk	Chen et al., 2012
		PRINCE	A global, network-based method for prioritizing disease genes and inferring protein complex associations	https://github.com/fosterlab/PrInCE	Vanunu et al., 2010
**Software**		Cytoscape	A software environment for integrated models of biomolecular interaction networks	https://cytoscape.org	Shannon et al., 2003
		Pajek	A tool for complex network analysis	http://mrvar.fdv.uni-lj.si/pajek	Dohleman, 2006


*These public databases are categorized into four types: TCM-related, drug-related, target-related, and disease-related. Publicly available tools and visualization software use data from these databases. Websites and references are provided for all of these resources.*

## Network Pharmacology Research and TCM

**FIGURE 2:**
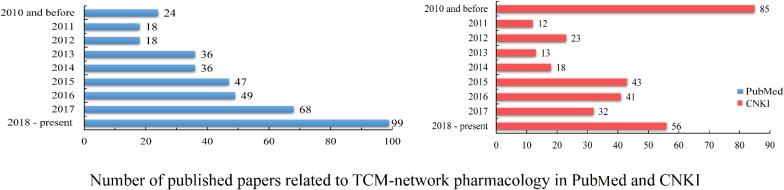
Published papers identified from PubMed and CNKI databases. Papers in PubMed were searched by Title/Abstract containing the following keywords: “network pharmacology,” “Chinese,” and “medicine.” Papers in CNKI were searched with the following key words: “network pharmacology” and “Chinese herb,” excluding all conference papers. The analysis time was from 2010 and before, to the end of 2018.

In recent years, an increasing number of studies have focused on the area of TCM network pharmacology. According to statistics in PubMed and the China National Knowledge Infrastructure (CNKI) databases, the number of published papers (Figure 2) has increased over time. These statistics demonstrate the increasing interest in the application of network pharmacology to TCM.

To date, network pharmacology has been applied to studies of many traditional Chinese herbs and herbal prescriptions. As listed in Table 2, researchers have conducted many network-based computational and experimental studies to detect effective substances and determine the mechanisms of herbal formulas against many diseases.

**Table 2:** TCM network pharmacology studies in international journals from 2011 to 2017.

Herb/Herbal prescription^a^	Related disease/effect	Year	Reference
*Aconiti Lateralis Radix*	Multi-targets	2011	Wu et al., 2011
Compound DanShen formula	Cardiovascular disease	2012	Li et al., 2012
Chinese herbal Radix Curcumae formula	Cardiovascular disease	2013	Tao et al., 2013
QingLuoYin	Rheumatoid arthritis	2013	Zhang et al., 2013
TaoHongSiWu decoction	Osteoarthritis	2013	Zheng et al., 2013
*Eucommia ulmoides Oliv.*	Hypertension	2014	Li et al., 2014d
GeGenQinLian decoction	Type 2 diabetes	2014	Li et al., 2014a
BuShenHuoXue formula	Chronic kidney disease	2014	Shi et al., 2014
LiuWeiDiHuang pill	Colon cancer and esophagitis	2014	Li et al., 2014b
QiShenYiQi formula	Myocardial infarction	2014	Li et al., 2014c
QiShenYiQi formula	Acute myocardial ischemia	2014	Wu et al., 2014
Dragon’s blood tablet	Colitis	2014	Xu et al., 2014
ZhiZiDaHuang decoction	Alcoholic liver disease	2015	An and Feng, 2015
Rhubarb	Renal interstitial fibrosis	2015	Xiang et al., 2015
SiMiaoWan	Gout	2015	Zhao et al., 2015
ErXian decoction	Menopause-related symptoms	2015	Wang S. et al., 2015
QiGuiTongFeng tablet	Gout	2015	Ke et al., 2015
*Salvia miltiorrhiza*	Cardiovascular disease	2015	He et al., 2015
SiNiSan	Depression	2015	Wang H.H. et al., 2015
ShengMai preparations	Cardio-cerebral ischemic diseases	2015	Li F. et al., 2015
*Dendrobium nobile*	Type 2 diabetes	2015	Li M.M. et al., 2015
MaHuangFuZiXiXin decoction	Allergic rhinitis	2015	Tang et al., 2015
XiaoYaoSan	Depression	2015	Gao et al., 2015
*Eriobotrya japonica* - *Fritillaria usuriensis* dropping pills	Pulmonary diseases	2016	Tao et al., 2016
XiaoYao powder	Anovulatory infertility	2016	Liu et al., 2016
*Euphorbia kansui* and *Glycyrrhiza*	Hepatocellular carcinoma ascites	2016	Zhang et al., 2016
QingFeiXiaoYanWan	Acute lung inflammation	2016	Hou et al., 2016
YinChenHaoTang	Severe acute pancreatitis	2016	Xiang et al., 2016
YinHuangQingFei capsule	Chronic bronchitis	2016	Yu et al., 2017
YangHe decoction	Breast cancer	2017	Zeng and Yang, 2017
*Fructus Aurantii Immaturus*	Anti-coagulation Gastrointestinal motility regulation activities	2017	Tan et al., 2017
SheXiangBaoXin pill	Cardiovascular disease	2017	Fang et al., 2017
Radix *salviae miltiorrhizae*	Hepatoprotective effect	2017	Hong et al., 2017


*^*a*^The biological ingredients of herbal prescription were listed in Supplementary Table S1.*

### Compatibility of TCM Ingredients

Traditional Chinese Medicine preparation (in other words, TCM prescription) is the main form of TCM, and it is produced under the guidance of TCM syndrome differentiation and treatment, which is highly scientific. The traditional “King, Vassal, Assistant, and Delivery servant” combination rule of TCM herbal formulas contains the rich and profound scientific connotation of TCM theory. Thus, the ability to explain the compatibility of TCM is one of the most challenging tasks for TCM research. Li et al. (2010) proposed a method named the Distance-Based Mutual Information Model (DMIM) to identify useful relationships that exist among herbs in numerous herbal formulas. DMIM combines mutual information entropy and “between-herb-distance” to score herb interactions and construct an herb network. It achieves a good balance among the herb’s frequency, independence, and distance in herbal formulas when used to retrieve herb pairs. They used 3,865 collateral-related herbal formulas to construct an herb network and illustrated the traditional herbal pairing and compatibility. LWDHW was used as an example to identify possible compatibility mechanisms, and it was found that LWDHW-treated disease shows high phenotype similarity and that certain “co-modules” are enriched in cancer pathways and neuro-endocrine-immune pathways, which may be the mechanism of action by which the same LWDHW formula treats different diseases. Zhang et al. (2008, 2017) focused on the five flavors of Chinese medicinal properties and constructed Bayesian network models of bitterness, pungent flavor, and sugariness. The five flavors of some TCMs were predicted by these established models, providing crucial support for research on TCM compatibility.

### Target Prediction of TCM

Understanding the molecular basis of TCM is crucial for the modernization of TCM, which not only would provide full theoretical support for TCM research but also would increase acceptance of TCM worldwide. In recent years, researchers have done a lot of work to study the molecular mechanisms of TCMs. We proposed the TCM-Mesh system (Zhang et al., 2017), which was designed as an integration of a database and a data-mining system for network pharmacology analysis of TCM preparations. We used TCM-Mesh to identify candidate targets for ginseng and LWDHW based on existing clues (e.g., molecular structures, bioactivity) from curated databases along with a pharmacology approach. The results showed that dammarane-type tetracyclic triterpenoids from ginseng (i.e., ginsenoside Rg1, ginsenoside Re, and ginsenoside Rb1) might be used to treat Alzheimer’s disease, hypertension, and atherosclerosis by targeting tumor necrosis factor (TNF), nitric oxide synthase 3 (NOS3), and AKT serine/threonine kinase 1 (AKT1). Chemical constituents from LWDHW (i.e., loganin, paeonol) can be used to treat diabetes, diabetic nephropathy, and diabetic retinopathy by targeting intercellular adhesion molecule 1 and connective tissue growth factor. Li et al. (Liang et al., 2014b) established a novel pharmacology approach based on a network pharmacology approach to analyze the traditional herbal formulas for LWDHW. The authors found that the compounds of LWDHW may play an important role in esophagitis and colon cancer by regulating the expression of C-C chemokine receptor 2 (CCR2), estrogen receptor 1 (ESR1), peroxisome proliferator-activated receptor gamma (PPARG), and retinoic acid receptor alpha (RARA). They also selected a XinAn medical family’s anti-rheumatoid arthritis (RA) herbal formula “QingLuoYin” (QLY) as an example (Zhang et al., 2013), which consisted of four herbs: KuShen (*Sophora flavescens*), QingFengTeng (*Sinomenium acutum*), HuangBai (*Phellodendron chinensis*), and BiXie (*Dioscorea collettii*). Some anti-angiogenic and anti-inflammatory active ingredients, such as kurarinone, matrine, sinomenine, berberine, and diosgenin, were identified among the 235 ingredients of QLY. In addition, the synergistic effects of the major ingredients were identified in QLY, such as matrine and sinomenine, which may have been derived from the feedback loop and compensatory mechanisms by targeting TNF- and vascular endothelial growth factor-induced signaling pathways involved in RA. According to the molecular structures of proteins and the corresponding targets of 1,401 drugs approved by the U.S. Food and Drug Administration, Wu et al. (2011) constructed a target prediction model by using the “Random Forest” algorithm. The authors used the compounds of “fuzi” (*Aconiti Lateralis Radix Praeparata*) to predict the targets and construct a multi-target network. The study found that the 22 compounds of “fuzi” have been used to predict several targets that reflect the characteristics of TCM. In addition, Li et al. (An and Feng, 2015) studied the antioxidant effects of ZhiZiDaHuang Decoction (ZZDHT) based on a network pharmacology approach. The authors found that ZZDHT may exert antioxidant effects to regulate reactive oxygen species, thereby treating alcoholic liver disease by targeting cytochrome P450 2E1 (CYP2E1), xanthine dehydrogenase (XDH), nitric oxide synthase 2 (NOS2), and prostaglandin-endoperoxide synthase 2 (PTGS2).

### Network Toxicology

Network toxicology is based on an understanding of the “toxicity-(side effect)-gene-target-drug” interaction network, which utilizes network analysis to speculate and estimate the toxicity and side effects of drugs. Liu et al. (2012) proposed that technologies that feature rapid preparation, high-throughput screening, toxic components exclusion, and biochip along with the drug-target network are of great importance to TCM research on active ingredient screening, toxic components exclusion, and molecular mechanism, which could increase the safety of TCMs. Fan et al. (2011) proposed the concept and framework of network toxicology of TCM. The related tools and technologies were briefly introduced, and the prospects for network toxicology of TCM were forecasted. In their work, they used network pharmacology methods to reconstruct the network of “compound-protein/gene-toxicity” to identify toxic substances and predict the toxic side effects of known compounds, which provides valuable information for understanding the toxic mechanisms. Currently, the related databases for the study of network toxicology includes CTD (Davis et al., 2011), TOXNET (Wexler, 2001), and the National Toxicology Program. Furthermore, additional foresting toxicity software, such as TOPKAT, HazardExpert, and DEREK (Wolfgang and Johnson, 2002), are available for TCM network toxicology studies.

## Pros and Cons of Current TCM Databases: Benchmarking

### Comparisons Among TCM Databases

**FIGURE 3:**
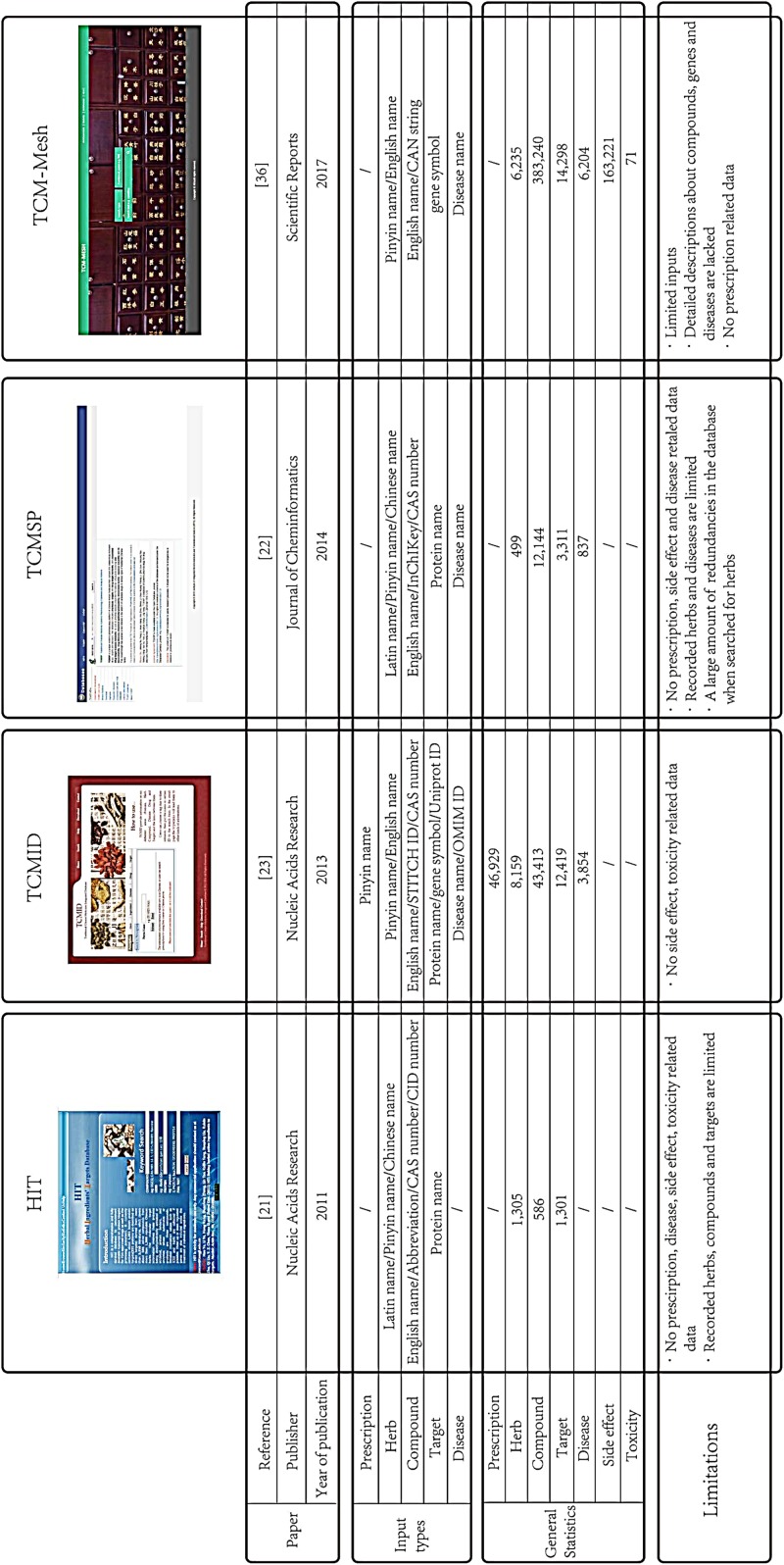
Comparisons among the four TCM databases. The comparison contains the paper information, different input types, general statistics, and limitations of several TCM databases.

As discussed, various databases are used for TCM analysis. However, network analysis of TCM is limited by several aspects of the currently available databases (Figure 3, current statistics on various databases can be obtained from the homepage of each database).

For example, HIT is a comprehensive TCM database that supports many different input formats. It is limited, however, in that the total information from HIT for TCM analysis is not sufficient, as it only contains about 586 compounds and 1,301 targets. Thus, the data that can be collected from the HIT are limited. TCMSP is a TCM database that captures the relationships among drugs, targets, and diseases. It allows users to use many input formats; however, TCMSP should be more concise as the database has a large amount of redundancies when searching for herbs, which must be trimmed to be improved. TCMID is another comprehensive database that provides information and bridges the gap between TCM and modern life science. It collects TCM-related information from the TCM@Taiwan database and the literature, including prescription of TCM, and also offers a module for network visualization. Although TCMID is available for multiple inputs, including compound name, STITCH database ID, and CAS number, when searching for ingredients, it is not sufficiently comprehensive, and many compounds are searched without a related network in TCMID. The newly launched TCM-Mesh is a more comprehensive database that not only contains the data on network pharmacology, including the “herb-compound-target-disease” network, but also includes data on TCM side effects and toxicity. As a result, TCM-Mesh offers a more holistic perspective for those conducting TCM research. Problems in TCM-Mesh remain to be resolved, however; for example, the web service is limited and many functions need to be improved, as the CAS number query is not supported. In addition, detailed descriptions about the compounds, genes, and diseases are lacking.

### Detailed Information on Herbs and Prescriptions for Assessment

We used prescriptions for LWDHW and ZZDHT to assess TCM-Mesh and randomly selected 10 well-known herbs to test different TCM databases for systematic assessment (Table 3).

**Table 3:** Detailed information on the data sets of herbs and prescriptions.

Herb or prescription	Index of assessment	Source
LWDHW	Targets	Liang et al., 2014b
ZZDHT		An and Feng, 2015
*Ganoderma lucidum*	(i) Number of ingredients of herbs found in the database	
Ginseng	(ii) Number of ingredient-target found in the database	Famous Chinese herbs
*Codonopsis pilosula*	(iii) Number of target-disease association found in the database	
*Astragalus membranaceus*		
Chinese yam		
Pseudo-ginseng		
*Polygonum multiflorum*		
Radix Angelicae dahuricae		
*Coptis chinensis*		
*Cordyceps sinensis*		


### Assessment Using LWDHW as an Example

As mentioned earlier, Li et al. (2010) found that the compounds of LWDHW may play an indispensable role in esophagitis and colon cancer by regulating the expression of CCR2, ESR1, PPARG, and RARA. To validate the effects of LWDHW on esophagitis and colon cancer, we used TCM-Mesh. We found 11 candidate targets (including PPARG) for colon cancer and four potential targets for esophagitis (Table 4), all of which were validated by the literature.

**Table 4:** Candidate targets of LWDHW for colon cancer and esophagitis validated by TCM-Mesh.

Disease	Target	Related compounds (herbs)	Reference
Colon cancer	ACP1 (acid phosphatase 1)	Adenine (*Poria cocos*)	Spina et al., 2008
	EGFR (epidermal growth factor receptor)	Capsaicin (Poria cocos), Cholesterol (Chinese yam)	Press et al., 2008
	GSTM1 (glutathione S-transferase mu 1)	Methyleugenol (cornel)	Huang et al., 2006
	IL10 (interleukin 10)	Dopamine (Chinese yam)	Cacev et al., 2008
	IL1B (IL-10b protein precursor)	Capsaicin (Poria cocos)	Lurje et al., 2009
	IL1RN (interleukin 1 receptor antagonist)	Palmitate (Poria cocos, Chinese yam, cornel)	Lurje et al., 2009
	PIK3CA (phosphatidylinositol-4,5-bisphosphate 3-kinase catalytic subunit alpha)	Palmitate (Poria cocos, Chinese yam, cornel)	Ogino et al., 2009
	PIK3R1 (phosphoinositide-3-kinase regulatory subunit 1)	Palmitate (Poria cocos, Chinese yam, cornel)	Li et al., 2008
	PPARG (peroxisome proliferator activated receptor gamma)	Capsaicin (Poria cocos)	Slattery et al., 2006
	RXRA (retinoid X receptor alpha)	Palmitate (Poria cocos, Chinese yam, cornel)	Egan et al., 2010
	SLC22A3 (solute carrier family 22 member 3)	Choline (Poria cocos), dopamine (Chinese yam)	Cui et al., 2011
Esophagitis	CYP2C19 (cytochrome P450 family 2 subfamily C member 19)	Capsaicin (Poria cocos), dopamine (Chinese yam), ursolic acid (cornel)	Kawamura et al., 2007
	GSTP1 (glutathione S-transferase pi 1)	Methyleugenol (cornel)	Kala et al., 2007
	IL1B (interleukin 1 beta)	Capsaicin (Poria cocos)	Koivurova et al., 2003
	IL1RN (interleukin 1 receptor antagonist)	Palmitate (Poria cocos, Chinese yam, cornel)	Koivurova et al., 2003


### Assessment Using ZZDHT as an Example

As discussed in Section 3.2, “Target prediction of TCM,” ZZDHT decoction has been applied to treat alcoholic liver disease by targeting CYP2E1, XDH, NOS2, and PTGS2. We used TCM-Mesh to validate the effects of ZZDHT, leading to the identification of 12 candidate targets (including CYP2E1) for alcoholic liver disease (Table 5), all of which were supported by the literature.

**Table 5:** Candidate targets of ZZDHT for alcoholic liver disease validated by TCM-Mesh.

Disease	Target	Related chemical composition (herbs)	References
Alcoholic liver disease	ADH1B (alcohol dehydrogenase 1B (class I), beta polypeptide)	Zinc (Cape jasmine)	Lorenzo et al., 2006
	ADH1C (alcohol dehydrogenase 1C (class I), gamma polypeptide)	Zinc (Cape jasmine)	Khan et al., 2010
	ALDH2 (aldehyde dehydrogenase 2 family member)	Manganese (Cape jasmine)	Yu et al., 2002
	CYP1A1 (cytochrome P450 family 1 subfamily A member 1)	Apigenin, genistein, quercetin (Cape jasmine), naringin, nobiletin, sinensetin, tangeretin, progesterone (rheum officinale)-	Burim et al., 2004
	CYP2E1 (cytochrome P450 family 2 subfamily E member 1)	Calcium (Cape jasmine), Geniposide (Cape jasmine)	Khan et al., 2009
	GSTM1 (glutathione S-transferase mu 1)	Zinc (Cape jasmine)	Ladero et al., 2005
	HFE (homeostatic iron regulator)	Calcium (Cape jasmine)	Ropero et al., 2001
	IL10 (interleukin 10)	Beryllium (Cape jasmine)	Gleeson et al., 2008
	IL2 (interleukin 2)	Beryllium (Cape jasmine)	Marcos et al., 2008
	IL6 (interleukin 6)	Chromium (Cape jasmine)	Gleeson et al., 2008
	PPARG (peroxisome proliferator activated receptor gamma)	Genistein (Cape jasmine)	Marcos et al., 2009
	XRCC1 (X-ray repair cross-complementing 1)	Chromium (Cape jasmine)	Rossit et al., 2002


### Comparison of Search Results Among the TCM Databases

To further compare the databases, we selected 10 well-known herbs to randomly evaluate the TCM databases: *G. lucidum*, ginseng, *Codonopsis pilosula*, *Astragalus membranaceus*, Chinese yam, pseudo-ginseng, *Polygonum multiflorum*, Radix *Angelicae dahuricae*, *Coptis chinensis*, and *Cordyceps sinensis*. We used each herb to search the different databases and compared the search results (Table 6).

**Table 6:** Search results of 10 well-known TCMs from three TCM databases.

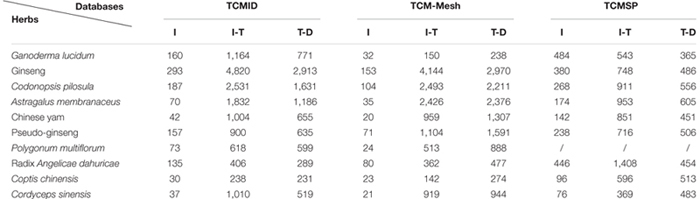

*I, Ingredients of herb; I-T, Ingredient-target associations; T-D, Target-disease associations.*

We compared the results from different databases and found that although the compound data recorded in TCM-Mesh were abundant, the data on the ingredients of the herb were limited. For each herb, the number of ingredients obtained from TCM-Mesh was lower than the number obtained from TCMID and TCMSP. We also found that the number of ingredients collected from TCMSP was much higher than that collected from the other two databases, in accordance with the fact that the database had a large amount of redundancies when searching for herbs. Additionally, we discovered higher completeness of TCM-Mesh in the ingredient-target associations and target-disease associations. For many herbs, the search results produced more hits in ingredient-target associations and target-disease associations in TCM-Mesh, although fewer ingredients were collected. Furthermore, a search for *P. multiflorum* in TCMSP produced no hits, revealing the limited herb data in TCMSP. The TCM database must keep pace with the development of TCM research to support the modernization of TCM. Therefore, a more comprehensive and complete database with powerful web services is necessary.

## Discussion and Conclusion

Research on TCM has a long history in China, illustrating the enormous potential and opportunities for new drug innovation. The inception of TCM network pharmacology offers researchers a new chance to gain systematic insights into TCM, as the research strategy of network pharmacology is in accordance with the holistic understanding of the effects of TCM on disease. This understanding may lead to a new direction for the research of pharmacological mechanisms and safety evaluation of TCM. In addition, related methods and studies must keep pace with the rapid developments in TCM research and must continue to be powerful.

### Unification and Integration: A More Powerful Database System

In recent years, several databases have been proposed, many of which share similar functions although they have different data sources. For most databases, the patterns of input differ from database to database. For example, the herb “Dried Tangerine Peel” in TCMSP must be searched by “chenpi” or “*Citrus reticulata*,” whereas in TCMID, it must be searched by “chen pi” or “*Citri reticulatae pericarpium*.” In addition, many compounds and proteins have different aliases in different databases (e.g., compounds: chemical name, CID number, STITCH ID, CAS number, PubChem CID, EC number, UNII; proteins: protein name, gene symbol, node ID, target ID, target drugbank ID, uniprot ID). The uniform format of input and output not only would make the current databases more concise, but also would enable researchers to take full advantage of the database resources. Thus, a standard transforming platform is needed. Such a platform should fully understand the data structure and content of various databases. Furthermore, it should support the translation among different formats of herbs, compounds, or proteins to bridge the gap between current databases for TCM research. Currently, the output of previous databases can be directly used as the input for another database. Most databases have associated web services; thus, the transforming platform that could transfer data from one database to another, can be presented to users as web services that are linked to different databases or to a browser plug-in. In addition to the unification of databases, the integration of diverse databases is also of great importance. Currently, more than 10,000 herbs used in more than 100,000 herbal formulas have been recorded in TCM (Qiu, 2007), posing a huge challenge for researchers who want to better understand the efficacy and the safety of the TCM. It is difficult for a single database to resolve all these problems as a result of the incompleteness of data. Each database has its own advantages regarding the specificity of its data and its algorithm. Therefore, the full integration of available resources will facilitate the production of an even more powerful and comprehensive database system, which undoubtedly will more effectively promote the modernization of TCM.

### The Content of Compounds: A Crucial Factor

TCM aims to restore whole-body balance in patients by using a herbal formula (Li et al., 2007), and herbal prescriptions are usually composed of two or more medical herbs in a certain proportion. For example, LWDHW consists of six herbs (Zhao et al., 2007; Xie et al., 2008): *Rehmannia glutinosa* libosch., *Cornus officinalis* Sieb., *Dioscorea oppositifolia* L., *Paeonia ostii*, *Alisma orientale* Juz, and *Poria cocos* (Schw.) *Wolf*, with a dose proportion of 8:4:4:3:3:3 (Wu et al., 2007; Wang et al., 2010). For current studies on TCM formulas, researchers focus on the chemical composition rather than on the proportion of each compound of TCM. Therefore, the influence of the compound content on the effect of the TCM is often ignored. The weight of the content of each compound should be taken into consideration in future studies to better understand the pharmacological effects of the TCM.

### TCM-Gut Microbiota Network Pharmacology: A New Frontier

The significant involvement of the gut microbiota in human health and disease suggests that manipulation of commensal microbial composition through combinations of probiotics, antibiotics, and prebiotics could be a novel therapeutic approach. Previous studies have shown that many TCMs can be used as agents to prevent gut dysbiosis (Chang et al., 2015; Zhou et al., 2016). Therefore, a more comprehensive database about TCM and gut microbiota is needed, which should not only include information on the interaction between the TCM and the gut microbiota but also integrate respective advantages of TCM databases and microbiome databases. This integration will be conducive to accelerating the internationalization of TCM and will allow researchers to fully understand the efficacy of TCM from a holistic perspective. Research in the area of TCM network pharmacology is still in its infancy and remains to be developed. However, with data accumulation on TCM and clinical research and interaction of various methods of analysis and experiment, researchers can obtain more substantive and authentic information. This information is conducive to the modernization and internationalization of TCM and may offer critical technological support for drug development, clinical diagnosis, and personalized medicine.

## Author Contributions

KN and HB conceived and proposed the idea. KN, HB, and RZ designed the work. KN, RZ, and XZ contributed to the interpretation of data for the work. KN, HB, and RZ drafted the work. KN, HB, XZ, and RZ revised it critically for important intellectual content. All authors read and approved the final manuscript to be published, and agree to be accountable for all aspects of the work in ensuring that questions related to the accuracy or integrity of any part of the work are appropriately investigated and resolved.

## Conflict of Interest Statement

The authors declare that the research was conducted in the absence of any commercial or financial relationships that could be construed as a potential conflict of interest.
