# Supplementary material for: Network Pharmacology Databases for Traditional Chinese Medicine: Review and Assessment
Source: Front Pharmacol. 2019 Feb 21;10:123. doi: 10.3389/fphar.2019.00123 (PMC6393382; doi:10.3389/fphar.2019.00123)
Supplement: Supplementary file 1 [file Table_1.DOCX]

**Supplementary material**

**Network Pharmacology Databases for Traditional Chinese Medicine: Review and Assessment**

*Runzhi Zhang, Xue Zhu, Hong Bai^*^, Kang Ning^*^*

*^1.^ School of Life Science and Technology, Huazhong University of Science and Technology, Wuhan, Hubei, 430074, China*

**Corresponding author: ningkang@hust.edu.cn, baihong@hust.edu.cn*

**Table S1. The biological ingredients contained of herbal prescription.**

| **Herbal prescription** | **Biological Ingredients** | **Reference** |
| --- | --- | --- |
| Compound Dan-shen formula | *Radix Salviae Miltiorrhizae*, *Panax Notoginseng*, and *Borneolum* | (1) |
| Chinese herbal *Radix Curcumae* formula | *Radix* *Curcumae*, *Fructus Gardeniae*, *Moschus*, and *Borneolum* | (2) |
| Qing-luo-yin | *Sophora flavescens*, *Sinomenium acutum*, *Phellodendron chinensis* and *Dioscorea collettii* | (3) |
| Tao-hong-si-wu decoction | *Radix Rehmanniae Preparata*, *Angelica sinensis*, *Radix Paeoniae Alba*, *Ligusticum chuanxiong*, *Prunus persica* and *Carthamus annuum* | (4) |
| Ge-gen-qin-lian decoction | *Puerariae Lobatae radix*, *Scutellariae radix*,  *Coptidis rhizoma*, and *Glycyrrhizae Radix* et  *Rhizoma Praeparata cum Melle* | (5) |
| Bu-shen-huo-xue formula | *Astragali radix*, *Curcumae rhizoma*, *Rheiradix et rhizoma*, *Cuscutae semen*, and *Salviae miltiorrhizae radix et rhizoma* | (6) |
| Liu-wei-di-huang pill | *Radix Rehmanniae Preparata*, *Fructus Corn*, *Rhizoma Dioscoreae*, *Rhizoma Alismatis*, *Cortex Moutan* and *Poriacocos* | (7) |
| Qi-shen-yi-qi formula | *Astragalus membranaceus*, *Salvia miltiorrhiza*, *Panax notoginseng*, and *Dalbergia odorifera* | (8) |
| Dragon's blood tablet | *Dracaena spp*., *Daemonorops spp*., *Croton spp*., and *Pterocarpus spp*. | (9) |
| Zhi-zi-da-huang decoction | *Gardenia jasminoides* Ellis, *Rheum officinale* Baill, *Citrus aurantium* L. , and Semen Sojae Preparatum | (10) |
| Si-miao-wan | *Phellodendron chinense* Schneid., *Atractylodes chinensis* (DC.) Koidz., *Achyranthes bidentata* Bl., *Coix lacryma-jobi* L. var. mayuen (Roman.) Stapf, *Smilax glabra* Roxb. (SG) and *Lonicera japonica* Thunb | (11) |
| Er-xian decoction | Herba *Epimedium Brevicornum*, Rhizoma *Curculiginis Orchioides*, Radix *Morindae Officinalis*, Radix *Angelicae Sinensis*, Cortex *Phellodendri Chinensis*, and Rhizoma *Anemarrhenae Asphodeloides* | (12) |
| Qi-gui-tong-feng tablet | *Astragalus membranaceus*, *Acanthopanar gracilistylus*, *Cinnamomum cassia*, *Fritillaria thunbergii*, *Artemisiae Anomalae*, *Piper kadsura*, *Fraxini Cortex*, *Dendranthema indicum* | (13) |
| Si-ni-san | Bupleuri Radix, Paeoniae Radix Alba, Aurantii Fructus Immaturus, *Glycyrrhizae* Radix et Rhizoma | (14) |
| Sheng-mai preparations | Ginseng Radix et Rhizoma, Ophiopogonis Radix, Schisandrae Chinensis Fructus | (15) |
| Ma-huang-fu-zi-xi-xin decoction | Herba Ephedrae, *Radix Aconiti Lateralis* and *Asarum heterotropoides* | (16) |
| Xiao-yao-san/ Xiao-yao powder | Radix Bupleuri, Angelicae Sinensis Radix, Poria Cocos (Schw.) Wolf., Paeoniae Radix Alba, Atractylodes Macrocephala Koidz., and Licorice | (17) |
| *Eriobotrya japonica* - *Fritillaria usuriensis* dropping pills | *Eriobotrya japonica* (Thunb.) Lindl., *Fritillaria usuriensis* Maxim., *Platycodon grandiflorum* (Jacq.) A. DC., *Pinellia ternata* (Thunb.) Makino. and volatile oil extracts from *Mentha haplocalyx* Briq | (18) |
| Qing-fei-xiao-yan-wan | Herba Ephedra, Gypsum Fibrosum, Pheretima, Fructus Arctii, Semen Lepidii, Bovis Calculus, Semen Armeniacae Amarum and Cornu Saigae Tataricae | (19) |
| Yin-chen-hao-tang | *Artemisia capillaris* Thunb., *Gardenia jasminoides* Ellis, and *Rheum officinale* Baill | (20) |
| Yin-huang-qing-fei capsule | *Ephedra sinica* Stapf, *Lepidium apetalum* Willd., *Semen Armeniacae Amarum*, *Fritillaria thunbergii* Miq, *Eriobotrya japonica* (Thunb.) Lindl., *Gypsum Fibrosum*, *Acorus tatarinowii* Schott, *Isatis indigotica* Fort., *Artemisia rupestris* L., *Dioscorea nipponica* Makino, *Schisandra chinensis* (Turcz.) Baill., *Ginkgo biloba* L, *Citrus aurantium* L., and *Glycyrrhiza uralensis* Fisch. | (21) |
| Yang-he decoction | *Rehmanniae Radix Praeparata*, *Cinnanmomi Cortex*, *Ephedra Herba*, *Colla Cornus Cervi*, *Sinapis Semen*, Licorice, *Zingiber officinale Roscoe* | (22) |
| She-xiang-bao-xin pill | Moschus, total ginsenoside ginseng root, Styrax, Cinnamomi Cortex, Bufonis Venenum, Bovis Calculus Artifactus and Borneolum Syntheticum | (23) |

**References**

1. Li X, Xu X, Wang J, Yu H, Wang X, Yang H, Xu H, Tang S, Li Y, Yang L, Huang L, Wang Y, Yang S. 2012. A system-level investigation into the mechanisms of Chinese Traditional Medicine: Compound Danshen Formula for cardiovascular disease treatment. PLoS One 7:e43918.

2. Tao W, Xu X, Wang X, Li B, Wang Y, Li Y, Yang L. 2013. Network pharmacology-based prediction of the active ingredients and potential targets of Chinese herbal Radix Curcumae formula for application to cardiovascular disease. J Ethnopharmacol 145:1-10.

3. Zhang B, Wang X, Li S. 2013. An Integrative Platform of TCM Network Pharmacology and Its Application on a Herbal Formula, Qing-Luo-Yin. Evid Based Complement Alternat Med 2013:456747.

4. Zheng CS, Xu XJ, Ye HZ, Wu GW, Li XH, Xu HF, Liu XX. 2013. Network pharmacology-based prediction of the multi-target capabilities of the compounds in Taohong Siwu decoction, and their application in osteoarthritis. Exp Ther Med 6:125-132.

5. Li H, Zhao L, Zhang B, Jiang Y, Wang X, Guo Y, Liu H, Li S, Tong X. 2014. A network pharmacology approach to determine active compounds and action mechanisms of ge-gen-qin-lian decoction for treatment of type 2 diabetes. Evid Based Complement Alternat Med 2014:495840.

6. Shi SH, Cai YP, Cai XJ, Zheng XY, Cao DS, Ye FQ, Xiang Z. 2014. A network pharmacology approach to understanding the mechanisms of action of traditional medicine: Bushenhuoxue formula for treatment of chronic kidney disease. PLoS One 9:e89123.

7. Liang X, Li H, Li S. 2014. A novel network pharmacology approach to analyse traditional herbal formulae: the Liu-Wei-Di-Huang pill as a case study. Molecular Biosystems 10:1014.

8. Li X, Wu L, Liu W, Jin Y, Chen Q, Wang L, Fan X, Li Z, Cheng Y. 2014. A network pharmacology study of Chinese medicine QiShenYiQi to reveal its underlying multi-compound, multi-target, multi-pathway mode of action. PLoS One 9:e95004.

9. Xu H, Zhang Y, Lei Y, Gao X, Zhai H, Lin N, Tang S, Liang R, Ma Y, Li D, Zhang Y, Zhu G, Yang H, Huang L. 2014. A systems biology-based approach to uncovering the molecular mechanisms underlying the effects of dragon's blood tablet in colitis, involving the integration of chemical analysis, ADME prediction, and network pharmacology. PLoS One 9:e101432.

10. An L, Feng F. 2015. Network pharmacology-based antioxidant effect study of zhi-zi-da-huang decoction for alcoholic liver disease. Evid Based Complement Alternat Med 2015:492470.

11. Zhao F, Guochun L, Yang Y, Shi L, Xu L, Yin L. 2015. A network pharmacology approach to determine active ingredients and rationality of herb combinations of Modified-Simiaowan for treatment of gout. J Ethnopharmacol 168:1-16.

12. Wang S, Tong Y, Ng TB, Lao L, Lam JK, Zhang KY, Zhang ZJ, Sze SC. 2015. Network pharmacological identification of active compounds and potential actions of Erxian decoction in alleviating menopause-related symptoms. Chin Med 10:19.

13. Ke ZP, Zhang XZ, Ding Y, Cao L, Li N, Ding G, Wang ZZ, Xiao W. 2015. Study on effective substance basis and molecular mechanism of Qigui Tongfeng tablet using network pharmacology method. Zhongguo Zhong Yao Za Zhi 40:2837-42.

14. Wang HH, Zhang BX, Ye XT, He SB, Zhang YL, Wang Y. 2015. Study on mechanism for anti-depression efficacy of Sini San through auxiliary mechanism elucidation system for Chinese medicine. Zhongguo Zhong Yao Za Zhi 40:3723-8.

15. Li F, Lv YN, Tan YS, Shen K, Zhai KF, Chen HL, Kou JP, Yu BY. 2015. An integrated pathway interaction network for the combination of four effective compounds from ShengMai preparations in the treatment of cardio-cerebral ischemic diseases. Acta Pharmacol Sin 36:1337-48.

16. Tang F, Tang Q, Tian Y, Fan Q, Huang Y, Tan X. 2015. Network pharmacology-based prediction of the active ingredients and potential targets of Mahuang Fuzi Xixin decoction for application to allergic rhinitis. J Ethnopharmacol 176:402-12.

17. Gao Y, Gao L, Gao XX, Zhou YZ, Qin XM, Tian JS. 2015. An exploration in the action targets for antidepressant bioactive components of Xiaoyaosan based on network pharmacology. Yao Xue Xue Bao 50:1589-95.

18. Tao J, Hou Y, Ma X, Liu D, Tong Y, Zhou H, Gao J, Bai G. 2016. An integrated global chemomics and system biology approach to analyze the mechanisms of the traditional Chinese medicinal preparation Eriobotrya japonica - Fritillaria usuriensis dropping pills for pulmonary diseases. BMC Complement Altern Med 16:4.

19. Hou Y, Nie Y, Cheng B, Tao J, Ma X, Jiang M, Gao J, Bai G. 2016. Qingfei Xiaoyan Wan, a traditional Chinese medicine formula, ameliorates Pseudomonas aeruginosa-induced acute lung inflammation by regulation of PI3K/AKT and Ras/MAPK pathways. Acta Pharm Sin B 6:212-21.

20. Xiang H, Wang G, Qu J, Xia S, Tao X, Qi B, Zhang Q, Shang D. 2016. Yin-Chen-Hao Tang Attenuates Severe Acute Pancreatitis in Rat: An Experimental Verification of In silico Network Target Prediction. Front Pharmacol 7:378.

21. Yu G, Zhang Y, Ren W, Ling D, Li J, Geng Y, Yi Z, Li D, Xu H, Yang H. 2017. Network pharmacology-based identification of key pharmacological pathways of Yin–Huang–Qing–Fei capsule acting on chronic bronchitis. International Journal of Chronic Obstructive Pulmonary Disease 12:85-94.

22. Zeng L, Yang K. 2017. Exploring the pharmacological mechanism of Yanghe Decoction on HER2-positive breast cancer by a network pharmacology approach. J Ethnopharmacol 199:68-85.

23. Fang HY, Zeng HW, Lin LM, Chen X, Shen XN, Fu P, Lv C, Liu Q, Liu RH, Zhang WD, Zhao J. 2017. A network-based method for mechanistic investigation of Shexiang Baoxin Pill's treatment of cardiovascular diseases. Sci Rep 7:43632.
